# Ikigai in aging hearts: Japan’s approach to cardiovascular care

**DOI:** 10.1038/s41440-025-02396-5

**Published:** 2025-10-17

**Authors:** Ryo Nakamaru, Akitoshi Hara, Yuji Yamada, Mitsuaki Sawano, Koichi Yamamoto

**Affiliations:** 1 Department of Cardiovascular Medicine, Kyorin University School of Medicine, Tokyo, Japan; 2 Department of Geriatric and General Medicine, The University of Osaka Graduate School of Medicine, Osaka, Japan; 3 Brookdale Department of Geriatrics and Palliative Medicine, Icahn School of Medicine at Mount Sinai, New York, NY USA; 4 Section of Cardiovascular Medicine, Department of Internal Medicine, Yale School of Medicine, New Haven, CT USA; 5 Center for Outcomes Research and Evaluation, Yale New Haven Hospital, New Haven, CT USA; 6 Teikyo Academic Research Center, Teikyo University, Tokyo, Japan

**Keywords:** De-implementation, Frailty, Geriatrics, Implemental hypertension

## Abstract

The global population is aging rapidly, creating a growing demand for cardiovascular care that is better aligned with the complex comorbidities and diverse life contexts of older adults. The 5Ms of geriatrics—Mind, Mobility, Medications, Multicomplexity, and What Matters Most—provide a practical framework to individualize treatment strategies based on functional status, comorbidities, and patient values (ikigai). This review explores how integrating the 5Ms into the management of hypertension, atrial fibrillation, and heart failure can guide clinical decision-making in aging societies. We highlight practical strategies, such as deprescribing, fall risk assessment, and shared decision-making, that tailor care to patient priorities. Furthermore, we discuss policy implications, including the need for greater incentives for comprehensive geriatric assessments and advance care planning. Since evidence-based guidelines may not fully capture the complexity of older patients, incorporating the 5Ms and comprehensive assessments can support patient-centered care and help build evidence based on meaningful outcomes in aging societies.

**Figure:**
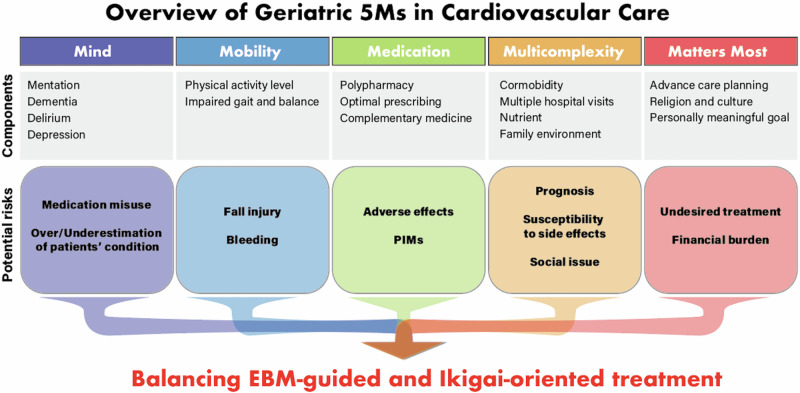
**Balancing EBM and Ikigai in Aging Hearts** The figure highlights how the 5Ms of geriatrics guides patient-centered decision-making. EBM evidenced based medicine

**Balancing EBM and Ikigai in Aging Hearts** The figure highlights how the 5Ms of geriatrics guides patient-centered decision-making. EBM evidenced based medicine

## Abstract (web summary)

Balancing EBM and Ikigai in Aging Hearts The figure highlights how the 5Ms of geriatrics guides patient-centered decision-making. EBM evidenced based medicine

## Introduction

Japan is at the forefront of global population aging, with an average life expectancy of 87.1 years for women and 81.1 years for men. Over 12.5 million individuals are aged ≥80, and ~7 million are classified as frail [1]. Many other Asian countries are also experiencing this dynamic demographic shift, with the aging population expected to increase significantly in the coming decades. This change in demographics presents profound challenges for cardiovascular disease (CVD) care, necessitating the development of more patient-centered healthcare models.

The 5Ms of geriatrics—Mind, Mobility, Medications, Multicomplexity, and What Matters Most—offer a concise yet comprehensive framework to address the varied needs of older adults.^2^ The final M, “What Matters Most” (or *ikigai* in Japanese) underscores personal values that make life worth living [2]. Given the regions’ rapidly aging populations, integrating the 5Ms into CVD care has the potential to promote individualized management and optimize outcomes. In this review, we illustrate how integrating the 5Ms may be able to guide individualized management of common CVDs––hypertension and its associated cardiovascular diseases, specifically atrial fibrillation (AF) and heart failure (HF)— with the goal of promoting care strategies that ensure these principles remain central throughout the care continuum (Graphical Abstract).

## Hypertension

Hypertension affects approximately three quarters of adults ≥75 and appears to be a natural consequence of aging rather than a disease in the oldest old [3]. On the other hand, it remains one of the most significant modifiable risk factor for cardiovascular diseases. The latest guideline from the European Society of Cardiology generally recommends strict BP control (<130/80 mmHg) even in older adults if tolerable [4]. Similarly, the recently published 2025 guidelines of the Japanese Society of Hypertension (JSH 2025) and the U.S. ACC/AHA hypertension guideline have adopted the same threshold as a treatment goal [5, 6]. However, unlike the other two guidelines, the JSH 2025 has dedicated an entire chapter on BP control for older adults underscoring the importance of careful assessment and monitoring of treatment tolerability in older adults. In the JSH 2025 guideline, clinicians are encouraged to consider balancing the therapeutic benefits of antihypertensive interventions against the potential risks of adverse events. In this context, incorporation of the 5Ms of geriatrics offers a standardized approach that can be readily implemented in everyday clinical decision-making.

### Mind/mobility

Cognitive impairment and reduced mobility can complicate blood pressure (BP) monitoring and adherence, increasing the risk of treatment-related adverse events, particularly orthostatic hypotension and falls that can lead to severe trauma and prolonged hospitalizations. Therefore, proactive cognitive screening and fall risk assessments such as an active standing test prior to BP control intensification can help determine whether older adults can safely maintain strict BP targets or would require individualized adjustments. The Japanese Cardiovascular Health Study (J-CHS) criteria have been designed to assess frailty through recent weight changes, handgrip strength, fatigue, gait speed, and engagement in daily physical activity, which allows categorization to three levels of physical fitness: robust, prefrail, or frail. In patients with frailty or higher levels of care dependence, particular caution is warranted regarding the tolerability of antihypertensive therapy [7].

### Medications

Achieving optimal BP levels in older adults often requires two or more antihypertensive agents, which inherently increases the risk of polypharmacy and its associated complications. A thorough medication review must consider not only the direct adverse effects of antihypertensive agents, such as renal dysfunction, electrolyte imbalances, and edema, but also the presence of potentially inappropriate medications (PIMs). Geriatricians commonly use the American Geriatrics Society Beers Criteria or STOPP/START criteria to reduce PIMs [8, 9]. More recently, anticholinergic burden—the cumulative effect of taking multiple medications with anticholinergic properties—has been recognized as a significant risk factor for frailty, delirium, falls, and mortality [10, 11]. Several tools have been developed to assess anticholinergic risk, most of which include antipsychotic and gastrointestinal agents. The Japanese Anticholinergic Risk Scale, created using expert consensus, lists 158 drugs, including important cardiovascular medications, and can help guide deprescribing [12, 13].

### Multicomplexity

Older adults with multi-comorbidities face higher risks of adverse effects from antihypertensive therapy. Additionally, social and economic factors that shape the caregiving environment play a crucial role in treatment feasibility. Depending on the patient’s cognitive status and caregiving circumstances, simplified medication regimens –such as once-daily dosing–may improve adherence and safety. Furthermore, older adults living alone may require heightened attention to the risk of falls. Accounting for both medical and social determinants is essential for prioritizing interventions that offer the highest immediate benefit.

### What matters most

Since antihypertensive therapy aims not only to lower blood pressure but also to prevent future cardiovascular events in both primary and secondary prevention settings, it raises a fundamental question: Is the primary goal extending survival at all costs, or is it to prioritize *ikigai*—a sense of purpose—even if that entails trade-offs in prognostic benefit? One important consideration is the lag time to benefit—the period required to prevent incident cardiovascular events—which is especially relevant when managing hypertension in older adults with limited life expectancy [14]. Engaging older adults in shared decision-making by asking about their *ikigai*, daily well-being, and willingness to accept side effects helps refine the blood pressure target. Further studies on cardiovascular prevention in late life are necessary to establish an evidence-based “less is more” approach that enhances well-being and quality of life [15].

## Atrial fibrillation

### Hypertension in AF: risk and management

Hypertension is a well-established risk factor for both the onset of AF and subsequent cardiovascular events in patients who already have AF [16]. Thus, optimal BP control is integral for AF management to reduce these risks. In a secondary analysis of the SPRINT (Systolic Blood Pressure Intervention trial), intensive blood pressure lowering reduced the risk of incident AF by ~25% [17]. Moreover, the effects were consistent across different age groups (<75 and ≥75 years). Nevertheless, in older adults—particularly those with clinically significant frailty—it is necessary to recognize the risks of BP reduction and AF management through the framework of the 5Ms of geriatrics, and to redefine clinical goals appropriately [4, 5].

### Mind/mobility

The presence of cognitive impairment and mobility limitations complicates decision-making regarding the initiation of anticoagulation therapy in frail patients. Among patients with severe frailty or advanced dementia with a prognosis of less than one year, de-escalating or even avoiding anticoagulation may be a reasonable option to prioritize patient comfort, representing a key aspect of shared decision-making [18, 19]. Meanwhile, it is essential to recognize that fall risk can be modified by underlying contextual factors. Rather than withholding anticoagulation solely due to fall risk, physicians should actively intervene to mitigate reversible fall risk factors such as the presence of PIMs, the anticholinergic burden, and muscle weakness. Routine risk assessment at every healthcare encounter and implementing fall prevention strategies are crucial to optimize stroke prevention while minimizing harm.

### Medications

In a regional registry of patients with AF, the proportion of older adults receiving oral anticoagulants (OACs) has increased from 41.3% in the pre- direct OAC (DOAC) era to 89.7% in the post-DOAC era [20]. This marked increase in DOAC use is driven by several factors, including physicians’ belief in improved safety, shorter half-life, simplified management without requiring frequent blood draws, and affordability in Japan relative to the traditional vitamin K antagonists. The ELDERCARE-AF (the Edoxaban Low-Dose for Elder Care Atrial Fibrillation Patients) trial demonstrated that very low-dose edoxaban effectively reduced systemic embolism without increasing bleeding risk in Japanese adults ≥80, regardless of frailty [21]. However, the FRAIL-AF (Frail Atrial Fibrillation) trial revealed potentially higher bleeding risk when switching from a vitamin K antagonist to a DOAC in frail older adults [22], illustrating the nuanced interplay between guideline recommendations and real-world patient complexity.

Given the high prevalence of polypharmacy in older adults, medication management must extend beyond OAC alone. Concomitant use of antiplatelet agents or nonsteroidal anti-inflammatory drugs can lead to a higher bleeding risk. Regular medication reviews, careful deprescribing when appropriate, such as discontinuing unnecessary aspirin when not indicated, are crucial to optimize the safety and efficacy of OACs. Particularly, mitigating bleeding risk requires a comprehensive approach. Clinicians should focus on modifiable factors, including suboptimal BP control, to tailor stroke prevention strategies while minimizing harm in older adults with multimorbidity and frailty.

Another underrecognized potential hazard is the prescription of antiarrhythmic drugs (AADs) to older adults. Age-related physiological changes—including reduced hepatic blood flow, diminished renal clearance, increased body fat, and lower plasma protein levels—can prolong the half-life and enhance the effects of AAD (e.g., hypotension, bradycardia, and QT interval prolongation) [18]. Therefore, careful dose selection, vigilant monitoring, and attention to drug–drug interactions are essential. A patient-centered approach, balancing rhythm control benefits against toxicity risks, remains crucial, and non-pharmacological options such as catheter ablation should be considered when appropriate. Indeed, recent registry data from Japan suggest that older adults with frailty who underwent catheter ablation for AF experienced improvements in frailty markers, such as weight loss, walking speed, and fatigue [23].

### Multicomplexity

Understanding the interplay between AF and multimorbidity is crucial for devising effective healthcare strategies [24]. Older adults with AF often have multiple chronic conditions, complicating risk assessments for both thrombosis and bleeding, while also impacting functional status, medication adherence, and treatment preferences. A prior epidemiological study reported that over 90% of older adults (≥65 years) initiating OACs who died during a 3-year follow-up experienced neither stroke nor major bleeding [25]. These findings highlight that their risks extend beyond cardiovascular events, emphasizing the need to shift clinical conversations toward broader, patient-centered goals. Care discussions should address patients’ overall clinical complexity and align treatment options with their individual values and prognosis.

### What matters most

Patient priorities-aligned care, an evidence-based approach in multimorbidity settings, has been associated with reduced treatment burden and improved goal-concordant care [26]. For some older adults, avoiding hospitalizations resulting from bleeding events associated with anticoagulation—and thereby maintaining quality of life—may be prioritized over potential long-term benefits. Early discussions around personal values and goals are much needed to guide treatment choices.

## Heart failure

### Hypertension and HF: risk and management

Treatment for hypertension plays a key role in preventing the development of symptomatic and advanced-stage HF. In the STEP (Strategy of Blood Pressure Intervention in the Elderly Hypertensive Patients) trial, which enrolled adults aged 60–80 years with hypertension, intensive treatment (i.e., targeting a systolic BP of 110–129 mmHg) resulted in a lower incidence of acute decompensated HF (hazard ratio, 0.27) [27]. In contrast, while for patients with established HF, there is no compelling evidence to support a single optimal BP target [28], the current U.S. hypertension guidelines recommend a target systolic BP of <130 mmHg, regardless of systolic function status [6]. For older adults, particularly those with frailty or multiple comorbidities, BP management should be individualized, considering potential cardiovascular benefits, symptomatic relief, and the patient’s overall priorities. These principles align closely with the 5 Ms of geriatrics.

### Mind/mobility

Cognitive impairment may impair a patient’s capacity to recognize worsening symptoms or adhere to complex medication regimens. Similarly, frailty exacerbates exercise intolerance and functional decline, leading to further worsening mobility [29]. Given these challenges, multidisciplinary support, including caregiver involvement and cardiac rehabilitation, is essential for optimizing HF management.

### Medications

Recent advances in pharmacological therapies for heart failure (HF), such as sodium-glucose cotransporter 2 (SGLT2) inhibitors and glucagon-like peptide-1 (GLP-1) receptor agonists, have contributed to reducing cardiovascular events across a broad range of left ventricular ejection fractions [30, 31]. However, the efficacy and safety of these therapies in frail older adults remain uncertain, as this population is often underrepresented in pivotal clinical trials [32]. Additionally, adverse effects—such as genital infections associated with SGLT2 inhibitors or sarcopenia linked to GLP-1 receptor agonists—may disproportionately impact frail individuals [33]. While current guidelines recommend avoiding the discontinuation of HF-specific medications unless contraindicated [34], careful dose adjustment, close monitoring, and individualized treatment plans are essential in older adults with multimorbidity. Concerns have also been raised that trials primarily designed to demonstrate benefits may underreport potential harms in vulnerable populations [32]. Further research is warranted to clarify the impact of suboptimal dosing compared to non-use in frail older adults.

### Multicomplexity

Multicomplexity increases vulnerability to both pharmacological and procedural interventions, complicating decision-making in older adults with HF. Indeed, non-cardiovascular deaths driven by multimorbidity can offset cardiovascular benefits [35, 36], challenging conventional risk-benefit calculations. This is particularly relevant for structural heart interventions, such as transcatheter valve procedures, which can result in high out-of-pocket costs. While largely covered by Japan’s universal healthcare, these procedures can still impose financial burdens on caregivers and society. The return on investment is limited when performed on patients with a shorter life expectancy, especially those with multiple comorbidities. From an individual perspective, patients and their families must weigh the potential benefits of improved quality of life against the risks, financial strain, and post-procedural care demands. These highlight the importance of individualized care strategies. Comprehensive geriatric assessments and frailty screening can aid in identifying patients who are more likely to benefit from interventions versus those for whom a focus on quality of life may be more appropriate [37].

### What matters most

The trade-offs between longevity, symptom relief, and quality of life become increasingly complicated. In end-stage HF, when interventional options or advanced medical therapies offer diminishing returns, conversations about palliation and comfort measures become vital. In Japan’s cultural context—where euthanasia and physician-assisted death are not allowed by its legal framework—family consensus often influences the course of care, underscoring the importance of early advance care planning that respects a patient’s *ikigai*. Clinicians can facilitate these conversations by exploring the patient’s values with open-ended questions. For example: “As you think about the future, what is most important to you?” followed by, “What are your biggest fears or worries about your health?” These questions help uncover a patient’s priorities and define the boundaries of acceptable trade-offs, ensuring that the care plan truly honors their *ikigai*.

## Health policy measures to promote comprehensive geriatric assessment

Strict adherence to evidence-based guidelines across multiple conditions may not always align with the practical realities of caring for older adults with complex health needs. Furthermore, this can place an unsustainable burden on patients and families, highlighting the need for a more holistic approach and the importance of appropriate de-implementation. Nonetheless, in many healthcare systems worldwide, there is little financial incentive for time-intensive, non-interventional activities such as advance care planning, despite their clinical significance. Japan’s healthcare system is distinctive in that it provides specific incentives to encourage comprehensive evaluations and deprescribing. For example, a reimbursement of ~$5 is allocated for comprehensive functional assessment conducted at the time of hospital admission. Similarly, deprescribing initiatives are incentivized at around $20 per case, which has contributed to a reduction in the number of patients taking ten or more medications [3]. However, reimbursement for functional assessments is currently limited to hospital admission, and these financial incentives pale in comparison to the substantial payments granted for procedural interventions. Enhancing incentives for regular comprehensive assessments rooted in the 5Ms framework––cognitive screening (Mind), physical performance assessments (Mobility), medication reviews (Medications), social and environmental evaluation (Multicomplexity), and conversations about *ikigai* (What Matters Most)—may boost a clinical approach that prioritizes patient-centered outcomes. Evaluating *ikigai* and quality-of-life as alternative clinical endpoints, rather than focusing solely on survival, is of great importance. Survey data reported by patients and their families can provide more practical insights into geriatric care. Integrating perspectives from clinical communication with new advancements will support more tailored care approaches.

Meanwhile, conducting a fully comprehensive geriatric assessment is both time-consuming and often impractical in routine clinical settings. Moreover, not all patients are able to clearly articulate what matters most to them, and some may be reluctant to disclose their social circumstances. Hence, the continuous incorporation of the 5Ms framework into routine clinical conversations is essential for progressively updating patient information and fostering mutual trust between clinicians and patients.

## Conclusion

The principles encapsulated by the 5Ms of geriatrics can help refocus cardiovascular care on individual goals, functional status, and quality of life. In clinical situations not fully covered by disease-specific guidelines, clinicians, caregivers, and patients must collaboratively determine an acceptable balance between survival and *ikigai*. Structured discussions, realistic reimbursement policies, and robust patient-reported outcomes are pivotal in making geriatric CVD care more equitable and patient-centered. By integrating these elements, aging societies can better align advanced medical interventions with the holistic well-being and dignity of older adults.
